# A Novel Analysis via Micro-CT Imaging Indicates That Chemically Modified Tetracycline-3 (CMT-3) Inhibits Tooth Relapse after Orthodontic Movement: A Pilot Experimental Study

**DOI:** 10.1155/2019/3524207

**Published:** 2019-04-01

**Authors:** Giovanni Modesto Vieira, Denise Pinheiro Falcao, Sormani Bento Fernandes de Queiroz, Valthierre Nunes de Lima, Ricardo Bentes de Azevedo, Valdenize Tiziane, Heitor Moreno, Rivadavio Amorim

**Affiliations:** ^1^Department of Medical Sciences, University of Brasília (UnB), Campus Darcy Ribeiro, Asa Norte, Zip Code: 70910-90, Brasilia, DF, Brazil; ^2^Department of Surgery and Integrated Clinic, University of Aracatuba Dental School (UNESP), 1193 Jose Bonifacio Street, Zip Code: 16015-050, Araçatuba, SP, Brazil; ^3^Department of Genetics and Morphology, Institute of Biological Sciences, University of Brasilia (UnB), Campus Darcy Ribeiro, Asa Norte, Zip Code: 70910-90, Brasilia, DF, Brazil; ^4^Department of Medicine, Brasilia Children's Hospital Brasilia, St. de Habitações Individuais Sul QI 15 - Lago Sul, Zip Code 71680-603, Brasilia, DF, Brazil; ^5^Department of Farmacology, Campinas State University (FCM/UNICAMP), 126 Tessalia Vieira de Camargo Street, Zip Code: 6111, Campinas, SP, Brazil

## Abstract

**Objective:**

To evaluate the effect of chemically modified tetracycline-3 (CMT-3) and simvastatin on tooth relapse after orthodontic movement in rats using a novel analysis method employing high-resolution micro-CT (Micro-CT) images. In addition, the correlation between bone density and orthodontic relapse was also evaluated for each experimental group.

**Methods:**

Forty adult male Wistar rats had stainless steel springs installed on their left upper first molars in order to generate tooth movement for 18 days. After this initial period, the animals were divided into three groups: (1) 30 mg/kg of CMT-3; (2) 5 mg/kg of simvastatin; and (3) 0.5% carboxymethylcellulose, and each group was treated for 20 days. Micro-CT images were analyzed (conventional method and 3D reconstruction) on the 7th and 18th days following spring fixation and finally, 20 days after treatment either with CMT-3 or simvastatin (38th day). Bone mineral density (BMD) of the mesial and distal roots of the upper first molar was also analyzed.

**Results:**

The difference was statistically significant between the groups as to recurrence (*p*=0.048), and the post hoc test identified the value of *p*=0.007 between the control group and the CMT-3 group. Simvastatin was not able to inhibit tooth relapse. The bone mineral densities of both the mesial and distal roots were different between the three groups, after the 20th day of drug use (*p*=0001 and *p* < 0001).

**Conclusion:**

Our findings support the initial evidence that CMT-3 is able to prevent relapse after tooth movement. Future trials in humans should evaluate such treatment as a promising approach to preventing this common phenomenon.

**Clinical Relevance:**

Considering the results obtained, CMT-3 can be used to avoid relapse after tooth movement.

## 1. Introduction

Tooth relapse after orthodontic movement is a poorly understood phenomenon that occurs due to acute or chronic tissue reaction, and it has a great impact on clinical practice [1, 2]. Its etiology remains unclear; nonetheless, overstretching of the supra-alveolar connective tissue fibers is one of the most accepted theories, among others (i.e., late eruption of third molars) [3, 4].

In fact, degradation of the extracellular matrix due to metalloproteinases (MMPs) activation has been related to the pathophysiological process of tooth movement [5]. The activation of MMPs can promote bone/dental and periodontal tissue remodeling (resorption vs. tissue formation) [6–8]. In addition, an inverse association between tooth movement rate and bone density has been indicated and that less mineralized bone is remodeled more easily [9].

Fixed retention devices are usually indicated as a strategy to prevent relapse and provide an appropriate time for bone and periodontal remodeling [10].

However, these devices do not effectively inhibit the relapse process, especially because of the release and higher expression levels of MMPs after the tooth movement phase [11, 12]. The traditional methods used to assess the phenomenon of tooth relapse, which include the parameters of image molding and data transfer, have not been indicated as accurate and precise compared with the gold standard microtomography (Micro-CT) method. Micro-CT uses the same properties of conventional tomography, but in a smaller scale framework allowing for an exponential increase in the resolution of the images [13, 14].

The literature has researched the effects of some drugs on bone metabolism due to their potential anabolic and anti-inflammatory effects in order to prevent orthodontic relapse. One of these is simvastatin, an inhibitor of 3-hydroxy-3-methylglutaryl-coenzyme A, which acts by decreasing bone resorption, via increased production of osteoprotegerin (OPG), acting in the inhibition osteoclast differentiation and promoting bone anabolism [15–17]. In addition, it is a drug that can modulate production levels of the nitric oxide synthetase enzyme, which is responsible for the formation of nitric oxide [18]. Nitric oxide affects the recruitment and differentiation of osteoblasts and osteoclasts, as well as acting in the regulation of metalloproteinases activities [19].

Tetracyclines have been considered a useful group of drugs in periodontal therapy, based on their antimicrobial activity [20]. And, at the end of the 1980s, chemically modified tetracyclines (CMTs) without antimicrobial activity [21] were developed but act in the inhibition of metalloproteinases [22, 23]. CMT-3 (6-deoxy-6-demethyl-4-dedimethylaminotetracycline) is the most potent MMP inhibitor, besides acting in cytokine production [24].

Chemically modified tetracycline-3 (CMT-3) has not yet been investigated in the context of tooth relapse. However, it might represent a potential novel therapy due to its ability to inhibit MMPs activity and modulate the proinflammatory process [25–27].

Based on the above considerations, the aim of this study was to evaluate the effect of CMT-3 and simvastatin on tooth relapse after orthodontic movement. Both a conventional method and a novel Micro-CT method were used for the primary outcome assessment. In addition, we investigated the relationship between relapse and bone density in areas around the dental roots.

## 2. Materials and Methods

### 2.1. Sample and Experimental Design

Forty adult male Wistar rats (*Rattus norvegicus albinus*) of 4 months of age and weighing an average of 354 ± 33 grams were selected. The rats were kept in standardized conditions with feed and water ad libitum, at ambient temperature, and in a 12 hour light-dark cycle (6 : 00 am–6 : 00 pm). This study was performed according to the Guide for Care and Use of Laboratory Animals policies published by the National Institutes of Health, USA. The study protocol used was previously approved by the local IRB (Approval number 7087/2012).

The animals were subjected to orthodontic movement for 18 days. After this period, the animals were divided into three groups as follows: a control group receiving 0.5% carboxymethylcellulose (m/v) plus *N*-methylpyrrolidone (*n*=10); an experimental group receiving 30 mg/kg of CMT-3 with methylpyrrolidone as a vehicle (*n*=15); and another receiving 5 mg/kg of simvastatin with 0.5% carboxymethylcellulose (m/v) as a vehicle (*n*=15). The simvastatin solution (350 mg) was prepared via dissolution in distilled water, and CMT-3 (4 grams) was prepared in a 13.6 ml solution of *N*-methylpyrrolidone to 5% ethanol and administered daily by oral gavage for 20 days, starting the treatment immediately after the orthodontic tooth movement period. Images were collected using micro-CT methods to assess the tooth movement and bone densitometry 7, 18, and 38 days after installation of the springs.

### 2.2. Tooth Movement

Upper first molars on the left side were selected to undergo tooth movement for 18 days. After anesthetized, each animal received 0.006 × 0.022 inch stainless steel spiral springs (Morelli, São Paulo, Brazil), which were installed according to the previously proposed model [28]. With the help of a 0.14 mm ligature wire of 9 cm in length, one spring end was passed between the first and second molars, holding one end of the spring between the first molar and the upper left central incisor on the same side. The other end of the spring was fixed to the central incisor ipsilateral with the aid of two 0.25 mm stainless steel ligature wires fixed to the cervical region of the central incisor with photopolymerizable resin after acid etching. An initial force of 75 cN was measured by using a properly calibrated precision dynamometer (Correx, Switzerland), equivalent to 2 mm extra length, making up 6 mm total length of the springs, which were measured by using a drypoint compass during their installation [29]. After the initial activation, the orthodontic device received no further activation during the period of the study. The movement occurred toward the mesial surface of the right first molar and was the object of study, discounting the movement toward the palatal surface of the ipsilateral upper incisor.

### 2.3. Assessment of Tooth Movement

**Figure 1:**
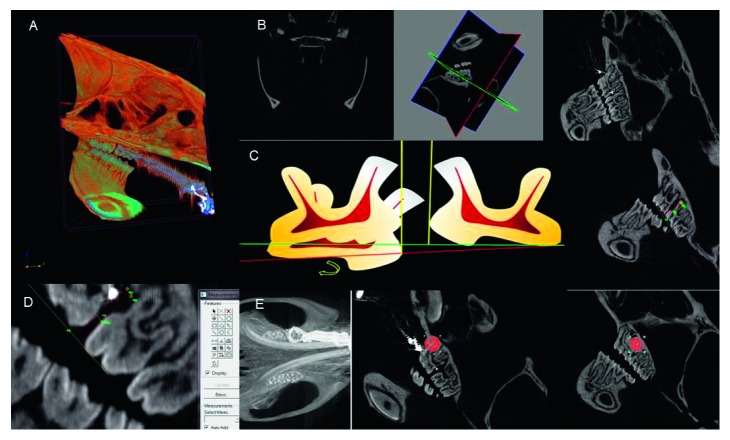
(a) Acquisition of Micro-CT image. (b) Reconstructed image selected using the *x*, *y*, and *z* system of coordinates. (c) Measuring guides: first, schema representing the extrusion and inclination of the first molars, whose measurement guide minimizes calibration errors between the molars; second, reconstructed image chosen with the measuring guides. (d) Measurement of tooth movement through reconstructed images. (e) Reconstructed Micro-CT image showing the circular area of 60 × 60 pixels in the mesial and distal roots of the left upper first molars for measurement of bone density in the delimited region.

The images were acquired with the aid of a Skyscan 1076 in vivo Bruker-Micro-CT scanner, generating micro-X-ray CT images (Figure 1(a)), which were later rebuilt with the Bruker-Micro-CT N-retraining 1.6.9.4 software (Figure 1(b)).

The acquisition time for Micro-CT images was 7 minutes for each specimen (100 kV, 1.0 mm AI filter, 35 *μ*m resolution). For the reconstruction of the images, we used the minimum value of 0.004915 and maximum value of 0.064752. The tooth movement assessment was performed by the Data Viewer version 1.5.0.0 software using 120 reconstructed micro-CT images. These images were used for the construction of measurement guides, and the image selection was performed using *x*, *y*, and *z* coordinates representing the following spatial planes: vertical, horizontal, and transversal (Figure 1(b)). The distal root of the first molar and the mesial root of the left second molar were assessed on the sagittal plane. This allows for a better evaluation due to (i) the sharpness of images along the entire length of the roots, (ii) visualization of three of the mice's molars on the same plane, and (iii) no artifact or overlapping of anatomical structures (Figure 1(b)).

The measurement guides were made using the Image Proc plus 5.1-Media Cybernetics software. Each guide was composed of a straight line representing the occlusal plane, originating at the tip of the mesiobuccal cusp of the first upper left molar and ending at the tip of the distobuccal cusp of the left second molar. Afterwards, two perpendiculars were made, tangential to the points of greatest convexity of the distobuccal root of the left first molar and mesiobuccal root of the left second molar. This procedure enabled the distance to be measured between these perpendiculars, minimizing measurement errors that normally occur due to the tilting motion and extrusion of the first molar [30] (Figures 1(c) and 1(d)).

### 2.4. Assessment of Bone Densitometry

Densitometry was performed using the Bruker-Micro-CT Analyser v.1.13 software. The regions of interest were selected by viewing the mesial and distal roots of the left first molar in the Micro-CT. The region of interest (ROI) consisted of a circle of 60 × 60 pixels, covering the mesial and distal roots and their surrounding alveolar bone structure, using this criterion for all the Micro-CT images analyzed (Figure 1(e)).

### 2.5. Statistical Analyses

The calculation of the sample revealed that at least 6 animals were needed in the control group and 10 animals in the experimental groups. Statistical analyses were performed using the Statistical Package for Social Sciences (SPSS® version 20.0 for Windows®, SPSS Inc./IBM Group, Chicago, USA). Continuous variables were expressed as mean or median (1st and 3rd quartiles) and standard deviation according to the data distribution assessed using the Kolmogorov–Smirnov test. The Kruskal–Wallis test followed by Dunn's post hoc test was performed to assess the relapse using the Micro-CT method, while ANOVA followed by Tukey's post hoc test was used to verify the weight and the bone mineral density (BMD) among the groups. Spearman's correlation test was used to test the relationship between bone density and orthodontic relapse. The level of significance accepted was 0.05 for relapse-related results, but *α* = 0.01 was specifically adopted for the BMD data.

## 3. Results

### 3.1. Analysis of Tooth Movement

**Figure 2:**
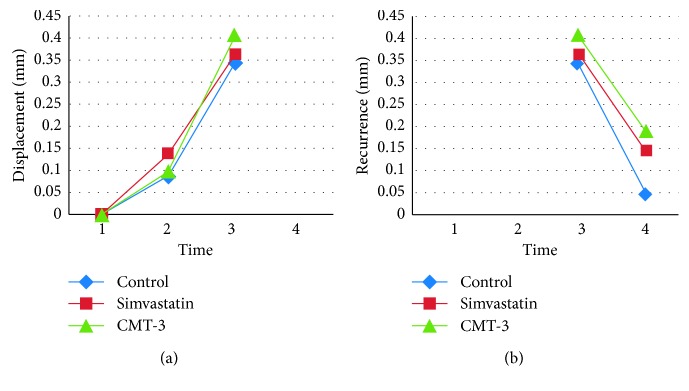
Graphs of displacement (a) and recurrence (b) of the tooth movement represented in millimeters in four different periods: (1) installation of springs, (2) 7 days after tooth movement, (3) 18 days after tooth movement and removal of the springs, (4) 38 days after tooth movement (20 days of drug regimen).

The tooth displacement was similar among the three groups compared, seven days (*p*=0.935) and eighteen days (*p*=0.16) after movement (Figure 2(a)).

The results, i.e., 38 days after tooth movement, (20 days of drug regimen) also showed that CMT-3 has the capacity to prevent the recurrence of tooth movement compared to the other groups (Figure 2(b)).

**Figure 3:**
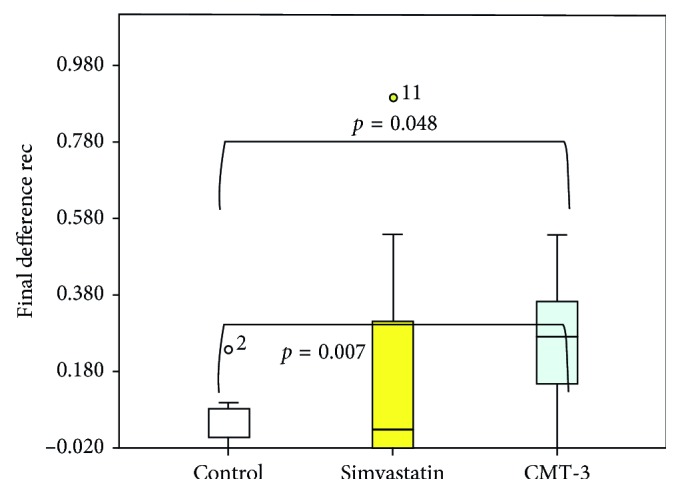
Difference in inhibition of recurrence of tooth movement between the three groups represented in millimeters by reconstructed images.

The difference in relapse was statistically significant between the three groups (*p*=0.048) (Figure 3).

The CMT-3-treated group showed inhibition of recurrence compared to the control group, through the post hoc test (*p*=0.007) (Figure 3). Moreover, no differences were found between the CMT-3 and simvastatin groups or between the simvastatin and control groups.

### 3.2. Analysis of Bone Mineral Density

**Figure 4:**
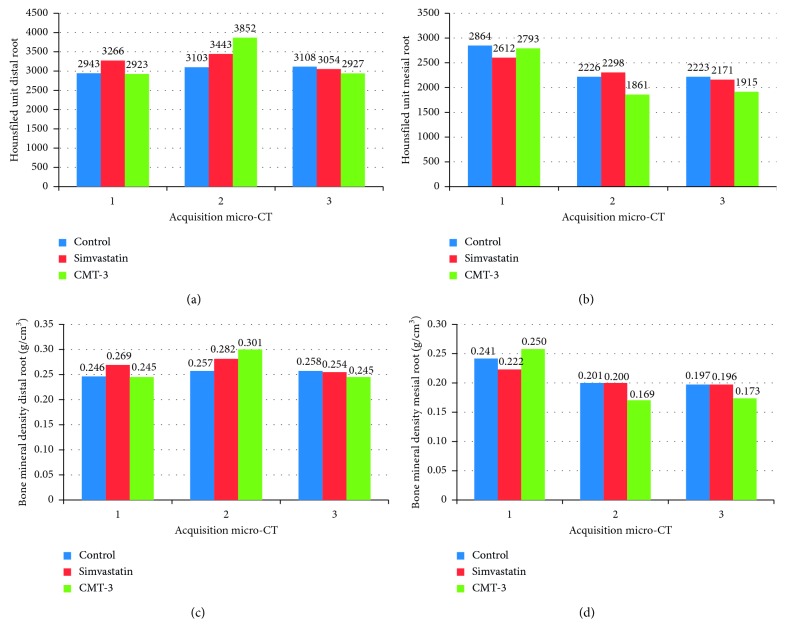
Graphs of Hounsfield units ((a) distal root and (b) mesial root) and bone mineral density (BMD, g/cm^3^) ((c) distal root and (d) mesial root): 1, seven days after tooth movement; 2, eighteen days after tooth movement; and 3, thirty-eight days after tooth movement (20 days of medication). (^∗^) Statistical difference between the CMT-3 group compared to the simvastatin group, control group, or both (simvastatin and control groups).

The bone mineral densities of both the mesial and distal roots were different (represented in Hounsfield units and BMD) between the three groups studied (Figure 4).

The CMT-3 group showed different final mesial and distal root densities from the other groups, after the 20th day of drug use (*p*=0.001 and *p* < 0.001), and Tukey's post hoc test demonstrated that the CMT-3 group was better when compared to the simvastatin and control groups. Finally, no correlation between bone density and orthodontic relapse was found for both distal and mesial roots (data not shown).

## 4. Discussion

The main finding of this study is the interesting inhibitory property of CMT-3 in tooth relapse after orthodontic movement. To our knowledge, this is the first scientific evidence of the use of CMT-3 for such situations. In addition, CMT-3 also increased the bone mineral density around the mesial root and decreased the density around the distal root. No association between bone density and orthodontic relapse was observed.

However, the results for CMT-3 are still important in this regard because they support its role in providing better stability and allowing for a proper remodeling process. Orthodontic recurrence is a major concern due to the bone metabolism and regeneration process after tooth movement. Hence, the development of new approaches to avoid or diminish this process could have a great impact on clinical practice.

Some studies have shown increased levels of various MMPs in gingival crevicular fluid from patients undergoing orthodontic treatment, indicating that these enzymes play a key role in bone remodeling [6, 7]. Considering our results, it can be suggested that the relapse inhibition provided by CMT-3 is related to the modulation of MMPs. The inhibitory effect of chemically modified tetracyclines (CMTs) on MMPs activity has already been described. One possible mechanism is the competitive inhibition of zinc, which affects the balance between MMPs and their natural inhibitory tissues (TIMPs) [26, 31, 32]. This effect may be due to structural differences in relation to conventional tetracycline and the removal of the dimethylamine group from position 4 of the ring, causing the loss of antimicrobial properties in the CMT-3 [33]. CMTs can also affect the recruitment of osteoclasts [34], by reducing MMP9, which is essential for the migration of these cells through the bone matrix [35] and promotes apoptosis of osteoclasts [36]. In addition, CMT-3 inhibits bone resorption, through anti-MMP and pro-TIMP actions, decreasing inflammatory cytokine action. CMT-3 can also directly inhibit the amidolytic activity of human leukocyte elastase (a serine proteinase) and the extracellular matrix degradation mediated by human leukocyte elastase [26, 37]. The pleiotropic properties of CMT-3 include the inhibition of serine proteinases, MMPs, and cytokines [26]. Therefore, CMTs cannot only be considered as a potential therapeutic strategy to inhibit dental relapse but also for other clinical applications in dentistry and medicine as well [38–40].

In our experiment, we expected the development of pressure areas on the mesial surface and tension areas on the distal surface in the upper right first molars. The mechanical stress imposed by orthodontic movement on the tension side generates an increase in anabolism with an increase in bone density. On the pressure side, the increased production of prostaglandins and interleukins promotes bone resorption, with decreased local bone density [41, 42]. After the orthodontic movement, we found a decrease in bone mineral density around the mesiobuccal root of the upper first molar, suggesting that the bone resorption in areas of pressure can precede the bone apposition in areas of tension.

In addition, during the period of tooth relapse, there was a slight increase in bone mineral density in the mesiobuccal root in the CMT-3 group compared to the control group. This finding may be related to the effect of CMT-3 in terms of inhibiting osteoclastic formation, decreasing bone resorption, and inducing osteoclast apoptosis [34, 43]. Another interesting point is that in the dental relapse stage, the CMT-3 group showed a decrease in bone density in the distobuccal root (*p*=0.01). This may indicate that the inhibitory effect of the drug on the tension side does not cause an imbalance in extracellular matrix remodeling. The bone mineral density in this root returned to values that were similar in magnitude to those prior to the dental movement.

Simvastatin is also involved in several mechanisms related to bone metabolism. It plays an important role in the anabolic phase and bone neoformation, enabling the expression of bone morphogenetic protein (BMP-2) and an increase in bone density [44]. Moreover, simvastatin also participates in the catabolic phase by regulating the differentiation of osteoclasts, with a possible interaction with MMP9, since this enzyme is essential for osteoclast recruitment [45]. Therefore, we hypothesized that simvastatin could also inhibit dental relapse. However, there was no significant difference in relapse between the simvastatin group and the control group. In addition, decreased bone density values in the region around the mesiobuccal root as well as the distobuccal root were found in the simvastatin group. In spite of the negative results found in this study, it should be mentioned that the dosage of simvastatin used (5 mg/kg) or the administration route could have had an impact on the primary outcome.

Additional studies with different dosages and alternative routes for simvastatin administration are needed to clarify this aspect.

Finally, the development of more accurate methods to assess the efficacy of drugs/devices to prevent tooth relapse is needed. In this regard, the use of Micro-CT is considered state of the art [13, 14]. In our study, the analyses of the Micro-CT images were performed using the conventional method and by 3D reconstruction [46]. Taken together, these analyses represent a strength of this study, especially because of the limitations related to experimental studies in rats (small anatomical structures, difficulty in performing conventional X-rays, etc.). In terms of better understanding the biological mechanisms of relapse and considering that the therapeutic prevention or control of this phenomenon would be of great value, our study might contribute to improving the devices currently used in the clinical setting, thereby implying more reliability for orthodontic practice.

## 5. Conclusion

Our findings support the initial evidence that CMT-3 is able to prevent relapse after tooth movement. Future trials in humans should evaluate such treatment as a promising approach to preventing this common phenomenon.

## Data Availability

The data can be found at http://repositorio.unb.br/handle/10482/18155 and https://doi.org/10.26512/2015.03.T.18155.
